# The Impact of SARS-CoV-2 Outbreak on Primary Sjögren's Syndrome: An Italian Experience

**DOI:** 10.3389/fmed.2020.608728

**Published:** 2020-12-07

**Authors:** Francesco Carubbi, Alessia Alunno, Claudio Ferri, Roberto Gerli, Elena Bartoloni

**Affiliations:** ^1^Internal Medicine and Nephrology Unit, Department of Life, Health and Environmental Sciences, Department of Medicine, University of L'Aquila, ASL 1 Avezzano-Sulmona-L'Aquila, L'Aquila, Italy; ^2^Rheumatology Unit, Department of Medicine, University of Perugia, Perugia, Italy

**Keywords:** COVID-19, primary Sjögren's syndrome, telemedicine, patient-reported symptoms, smart working

## Abstract

**Objective:** Since no data is available about the personal experience of people with primary Sjögren's syndrome (pSS) with regard to disease burden and management during the novel Severe Acute Respiratory Syndrome coronavirus (SARS-CoV)-2 outbreak, we aimed to explore these aspects with the ultimate goal to identify unmet needs and priorities.

**Methods:** A telephone consultation was scheduled with patients with pSS and information regarding the disease status, ongoing treatment and symptoms/diagnosis of coronavirus disease 2019 (COVID-19) were collected. Clinical records were retrospectively evaluated to gather pre-COVID-19 information.

**Results:** One hundred and two patients with pSS were contacted. Most rheumatology consultations and other pSS-related tests were canceled during the SARS-CoV-2 outbreak. Less than 30% of patients contacted the rheumatologist via telemedicine despite experiencing disease flares or therapy shortage. Disease activity and patient reported symptoms significantly worsened during the closure period. All patients practiced social distancing, most of those employed switched to smart working and different work settings impacted on the type of symptom worsening.

**Conclusion:** This is the first study addressing the personal experience of pSS patients resulting from the impact of the SARS-CoV2 outbreak and it identifies unmet needs and priorities requiring to be addressed. Our findings may help designing individualized strategies

## Introduction

The novel Severe Acute Respiratory Syndrome coronavirus (SARS-CoV)-2 outbreak represented a major challenge for the healthcare system worldwide and dramatically affected the management of people with chronic diseases, including rheumatic and musculoskeletal diseases (RMDs) (1–3). This was particularly pronounced in severely affected countries, such as Italy, undergoing total closure of out- and in-patient services in rheumatology and other specialties. In this setting, telemedicine rapidly became the predominant way for delivering care and remotely monitoring people with RMDs over the last months (4, 5). However, this abrupt change of the model of care, with telemedicine being imposed rather that mutually chosen by physicians and patients, revealed pitfalls and issues interfering with the care of people with RMD (6, 7).

The joint effort of rheumatologists across the world gave birth to collaborative initiatives aimed at mapping the incidence and outcomes of coronavirus disease 2019 (COVID-19) in people with RMDs^1^. The analysis of the first 600 COVID-19 cases reported from 40 countries outlined that the rheumatoid arthritis, systemic lupus erythematosus and psoriatic arthritis were the most frequent RMDs, while only 5% of COVID-19 cases were observed in patients with primary Sjögren's syndrome (pSS). The analysis did not reveal peculiarities with regard to any of the assessed variables (e.g., predictors of hospitalization) in pSS patients. However, in the overall cohort prednisone ≥10 mg/day or equivalent was associated with a higher odds of hospitalization compared with no steroid therapy (8).

To our knowledge no data is available about the personal experience of people with pSS as a consequence of the COVID-19 outbreak impact on the healthcare system, therefore this study aimed at addressing this gap in order to identify unmet needs and priorities.

## Materials and Methods

### Study Cohort

Patients with pSS according to the 2016 ACR-EULAR classification criteria (9) were contacted to schedule a telephone consultation. Information regarding the disease status, ongoing treatment and symptoms/diagnosis of COVID-19 were collected. Patients were asked whether they had performed exams again within the 10 days preceding the call. If not, the teleconsultation was rescheduled according in the subsequent days. Clinical records were retrospectively evaluated to gather clinical and therapeutic information as of the last face-to-face consultation before the closure of rheumatology services due to COVID-19. Disease activity was assessed with the EULAR Sjögren Syndrome Disease Activity Index (ESSDAI) (10) while patient reported symptoms were evaluated with the EULAR Sjögren's Syndrome Patient Reported Index (ESSPRI) (11) and with self-reported xerostomia and xerophtalmia on a 0–10 cm visual analog scale (VAS). All subjects provided informed consent as approved by local Institutional Review Board (ASL1 Avezzano Sulmona L'Aquila).

### Statistical Analysis

Data were analyzed with STATA/SE 16.1. The Wilcoxon matched pairs test was used to compare continuous variables while Fisher's exact test was used for categorical variables. All tests were two tailed and values of *p* < 0·05 were considered statistically significant. Values are reported as number (pergentage) or mean ± standard deviation (*SD*) throughout the manuscript.

## Results

One hundred and two pSS patients (99 females and three males) agreed to schedule the telephone consultation between 10 and 24th August 2020. Eight pSS patients could not be reached (not answering the phone/mobile phone switched off). Ninety-seven (95%) patients had their face-to-face rheumatology visit scheduled during the period of COVID-19 closure and it was canceled while the remaining five patients had the last face-to-face shortly after the lockdown and the subsequent face-to-face visit scheduled in summer shortly after we contacted them. As shown in Table 1, only 27 (26%) patients contacted their rheumatologist by telemedicine (either telephone or computer) during the closure and a disease relapse was the most frequent reason. In total, 23 (22%) patients experienced a disease relapse with articular manifestations being the most frequent [13 (56%) patients], followed by salivary gland fatigue [4 (17%) patients], salivary gland swelling [3 (13%) spontaneously changed their treatment regimen (in all cases a short CS course)]. Of the 39 patients taking HCQ, 16 (41%) experienced drug shortage and had to interrupt the treatment for a median of 20 days. Besides the shortage, 2 patients interrupted the treatment either as personal decision (*N* = 1) or upon suggestion of the general practitioner (GP) (*N* = 1) for the fear of being at higher risk of infection by SARS-CoV2.

**Table 1:** Personal experience of patients with primary Sjögren's syndrome with regard to disease status and management and the use of telemedicine during the COVID-19 total and partial closure.

Had their rheumatology consultation canceled	97 (95)
Had their laboratory tests/other tests related to pSS canceled	85 (83)
Contacted rheumatologist by telemedicine	27 (26)
**Reason for consulting rheumatologist by telemedicine**	
*Consultation for disease relapse*	13
*Consultation without disease relapse*	8
*Information on ongoing treatment (e.g., HCQ shortage) and/or COVID-19*	6
Changed treatment for disease relapse upon remote consultation with the rheumatologist	8/23 (35)
**Type of treatment change**	
*Dose increase or introduction of corticosteroids*	8/8 (100)
Changed treatment for disease relapse without remote consultation with the rheumatologist	5/23 (22)
**Type of treatment change**	
*Dose increase or introduction of corticosteroids*	5/5 (100)
Experienced HCQ shortage during the total/partial closure	16/39 (41)
Stopped treatment with HCQ because of drug shortage	11/16 (69)
Days without HCQ therapy before restocking, median (range)	20 (7–60)
**Interventions to minimize the duration of HCQ therapy interruption due the shortage**	
*No dose change, interruption when tablets over*	7/16
*Reduced daily dose from 400 to 200 mg*	3/16
*Reduced weekly dose taking 400 mg/day for 6 days per week*	1/16
*Had enough tablets to cover the period until restocking*	5/16
Ever considered to stop immunosuppressive therapy (not due to shortage)	2/47
**Reason for considering to stop immunosuppressive therapy**	
Fear of being infected by SARS-CoV2	1/2 (50)
Upon GP suggestion	1/2 (50)
Spontaneously stopped immunosuppressive therapy without consulting the rheumatologist	2/2 (100)
Experienced a disease relapse	23 (22)
Experienced one of the following symptoms: diarrhea, sore throat, non-productive cough, fever >37.5^°^C, reduced smelling or tasting, flu-like symptoms since the beginning of COVID-19 outbreak	13 (13)
Practiced social distancing according to government guidelines for the general population?	102 (100)
If employed, able to switch to smart working	26 (25)
Having used a face mask more than an average of 3 h daily	59 (58)
Having used a face mask more than an average of 3 h daily and having noticed a worsening a sicca symptoms	44/52 (85)^*^
Having used a face mask less than an average of 3 h daily and having noticed a worsening a sicca symptoms	12/50 (24)^*^
Having been in contact with a suspected or confirmed case of COVID-19	6 (6)
Having been diagnosed with COVID-19	2 (2)

*Numbers indicate the number of respondents (percentage) unless otherwise specified. The denominator is the total number of patients (N = 102) unless otherwise specified. SD, standard deviation, HCQ, hydroxychloroquine*.

* *p < 0.0001 calculated with Fisher's exact test*.

All patients reported having performed social distancing according to national guidelines and 52 (51%) patients reported use of a face mask for more than 3 h daily on average, and 44 of them (85%) noticed a worsening of the symptoms related to mucosal dryness. This proportion was significantly higher compared to that of patients using a face mask for <3 h daily (12/50, 24%; *p* < 0.0001).

Thirteen (13%) patients experienced symptoms that could be related to COVID-19 (e.g., sore throat, non-productive cough). All of them were tested for SARS-CoV2 infection by nasopharyngeal swab but only 2 had proven SARS CoV2 infection and were eventually diagnosed with COVID-19. Six patients (6%) were aware of previous contact with a suspected or confirmed case of COVID-19, most of them did not develop symptoms and the general practitioner advised asymptomatic patients to self-isolate for 14 days and contact the regional help-line only upon appearance of fever or other symptoms.

They 2 pSS patients with COVID-19 were females, aged 48 and 69 years with a disease duration of 4 and 10 years, respectively. The reported sicca symptoms at the last face to face consultation before the closure were similar (VAS dryness 4/10 and 5/10, VAS xerostomia 4/10 and 5/10; VAS xerophtalmia 4/10 and 5/10). However, the younger patient had more pronounced pain (VAS pain 8/10) and fatigue (VAS fatigue 8/10) compared to the older patient (VAS pain 5/10) and fatigue (VAS fatigue 5/10). Both patients were treated with HCQ and despite both of them experienced a disease relapse during the closure, only the older patients contacted the rheumatologist via telemedicine and she was advised to increase the dose of CS. The younger patient spontaneously started a short CS course. Of interest, none of the two patients was aware of previous contact with a suspected or confirmed case of COVID-19. The younger patient required hospitalization and was discharged after recovery without needing admission to intensive care unit. The older patient was managed at home and also had a favorable outcome.

In our cohort, disease activity, the VAS dryness, VAS xerostomia, VAS xerophtalmia and ESSPRI worsened during the closure compared to the last face to face consultation. Conversely, the VAS pain and VAS fatigue were similar (Table 2). In this regard, employed patients reported slightly different complaints according to the possibility or not to perform smart working. For example, those working from home often reported a worsening of xerophtalmia due to increased number of hours spent working on the computer, while patients who could not perform smart working (e.g., employed in stores selling essential goods), reported marked worsening of xerostomia and dry nose due to prolonged use of face masks. As shown in Table 3, a significant worsening of both xerostomia and xerophtalmia was observed in employed patients performing smart working while only xerostomia was significantly worsened in employed patients that had to be in the workplace and therefore use face masks for several consecutive hours.

**Table 2:** Disease activity and patient reported symptoms as of the last face to face rheumatology consultation before the closure of rheumatology services and at the date of telephone consultation.

	**Before closure**		**During closure**		*p-*value^*^
	**mean**	***SD***	**mean**	***SD***	
ESSDAI	4.5	3.9	5.0	4.1	0.041
ESSPRI	5.9	2.1	6.4	2.2	<0.0001
VAS dryness (global)	6.0	2.2	6.9	2.2	<0.0001
VAS xerostomia	6.1	2.5	7.2	2.5	<0.0001
VAS xerophtalmia	5.8	2.8	6.4	2.9	0.0002
VAS pain	5.5	2.9	5.9	2.9	0.062
VAS fatigue	6.3	2.8	6.5	2.7	0.067

*ESSDAI, EULAR Sjögren's syndrome disease activity index; VAS, visual analog scale*.

* *p-value was calculated with the Wilcoxon matched pairs test. ESSDAI values before closure were available in 71/102 (70%) patients. All the other variables were available in all 102 patients*.

**Table 3:** Changes in reported symptoms according to employment status and possibility to perform smart working during the COVID-19 related closure.

	**Employed and allowed to perform smart working (*****N*** **=** **30)**				*p*-value^*^	**Employed and not allowed to perform smart working (*****N*** **=** **11)**				*p*-value^*^
	**Before closure**		**During closure**			**Before closure**		**During closure**		
	**mean**	***SD***	**mean**	***SD***		**mean**	***SD***	**mean**	***SD***	
VAS dryness (global)	5.9	2.1	7.5	2.1	<0.0001	5.9	1.4	6.3	1.3	0.52
VAS xerostomia	5.9	2.2	7	2.6	0.0006	5.4	2.2	6.7	2.5	0.02
VAS xerophtalmia	5.6	2.7	7	2.6	0.04	6.4	1.8	6	1.8	0.41

*VAS, visual analog scale*.

* *p-value was calculated with the Wilcoxon matched pairs test*.

## Discussion

To the best of our knowledge this is the first study addressing the personal experience of pSS patients resulting from the impact of the SARS-CoV2 outbreak on the healthcare system, and including drug shortage, the use of telemedicine and the disease status during the closure of rheumatology services. In our cohort, COVID-19 was diagnosed in only 2 (2%) patients which is slightly lower than the data from international registries (8). Although both patients were taking prednisone >10 mg/day, which was identified as a risk factor for hospitalization, only one of them was admitted to the hospital. Surprisingly, the hospitalized patient was the one below the age of 65 despite older age was associated with higher odds of hospitalization (8).

A consistent number of patients experienced a disease relapse or HCQ shortage, but only a small proportion of them contacted the rheumatologist via telemedicine. In an historical period when telemedicine may become the rule rather than one of the possible healthcare options, patient empowerment and reassurance about the quality of care delivered by telemedicine is crucial to prompt people seeking help via these tools and to keep pursuing a shared decision-making model. This relies on a trustful physician patient relationship and has the ultimate goal to prevent negative outcomes and improve quality of life. Such approach is of particular importance during the SARS-CoV2 outbreak since: (i) prednisone ≥10 mg/day or equivalent was found to be associated with a higher odd of hospitalization in patients with RMDs and COVID-19 (8); (ii) there is still a lot of debate around the use of HCQ for prophylaxis and/or treatment of COVID-19, as well as ongoing trials on other compounds used to treat people with RMDs, hence future drug shortages cannot be excluded (12); (iii) it is not possible to foresee for how long social distancing and remote consultations may remain a necessity. In addition, isolation and social distancing may dramatically impact on psychological well-being of patients with RMDs (13). This may add a layer of complexity to remote management and underscores the importance to explore also the psychological status in order to capture a comprehensive picture of the individual's status even at distance. Based on the above-mentioned concepts, further research is needed to identify and implement best practice models for telemedicine in different RMDs (6). A systematic literature review aimed at assessing the effectiveness, acceptability and costs of telemedicine across different specialties yielded conflicting results and this reinforces the concept that specialty-specific or even disease specific telemedicine approaches are advisable (14). In our study access to (and knowledge of) technology did not emerge as barrier to the use of telemedicine. In this regard, the use of smartphone applications may improve the experience with telemedicine of pSS patients on one hand and ensure continuous monitoring of the disease on the other (15). Nevertheless, stakeholders developing such programs should carefully explore and take into account potential technical barriers that may affect implementation or acceptability by patients as recently emerged by a worldwide survey across rheumatologists (16).

The worsening of patient reported symptoms during the SARS-CoV2 outbreak in our cohort deserves to be appraised. Although the proportion of patients noticing a worsening of sicca symptoms was higher in the group wearing a face mask for longer periods during the day, this problem was reported by patients using this personal protective equipment for less time. All patients were aware of national regulations and recognized the importance of social distancing and the use of face masks^2^ and those performing smart working appreciated being given this opportunity. However, coping with the worsening of symptoms due to overuse of computers and face masks in the long term may become difficult or even overwhelming possibly leading to excessive self-isolation and even to the need to quit a paid job.

In conclusion, we acknowledge that the main limitation of our study is the low number of included patients, however, this is the first detailed assessment of the burden on SARS-CoV-2 outbreak on patients with pSS. In the uncertainty of COVID-19 trajectories in the future, the results of our study may help designing individualized strategies for telemedicine and holistic management of patients with pSS within the framework of novel models of care.

## Data Availability Statement

The original contributions presented in the study are included in the article/supplementary materials, further inquiries can be directed to the corresponding author/s.

## Ethics Statement

All subjects provided informed consent as approved by local Institutional Review Board (ASL1 Avezzano Sulmona L'Aquila).

## Author Contributions

All authors participated in the development of the project, the interpretation of the data, the manuscript preparation, and approved the current version of the manuscript.

## Conflict of Interest

The authors declare that the research was conducted in the absence of any commercial or financial relationships that could be construed as a potential conflict of interest.

## Footnotes

^1^The COVID-19 Global Rheumatology Alliance. Available online at: https://rheum-covid.org/.

^2^Advice on the use of masks in the context of COVID-19 from the World Health Organization. Available online at: https://apps.who.int/iris/bitstream/handle/10665/332293/WHO-2019-nCov-IPC_Masks-2020.4-eng.pdf.
